# Assessing the Feasibility of a Social Media to Promote Weight Management Engagement in Adolescents with Severe Obesity: Pilot Study

**DOI:** 10.2196/resprot.8229

**Published:** 2018-03-19

**Authors:** Elizabeth Prout Parks, Reneé H Moore, Ziyi Li, Chanelle T Bishop-Gilyard, Andrew R Garrett, Douglas L Hill, Yasmeen P Bruton, David B Sarwer

**Affiliations:** ^1^ Division of Gastreoenterology, Hepatology and Nutrition The Children's Hospital of Philadelphia Philadelphia, PA United States; ^2^ Department of Pediatrics Perelman School of Medicine University of Pennsylvania Philadelphia, PA United States; ^3^ Department of Biostatistics and Bioinformatics Emory University Atlanta, GA United States; ^4^ Biostatistics Collaboration Core Emory University Atlanta, GA United States; ^5^ Department of Psychiatry The Children's Hospital of Philadelphia Philadelphia, PA United States; ^6^ College of Science and Technology Temple University Philadelphia, PA United States; ^7^ Pediatrics Advanced Care Team The Children's Hospital of Philadelphia Philadelphia, PA United States; ^8^ College of Public Health Temple University Philadelphia, PA United States

**Keywords:** e-medicine, adolescents, social media, obesity, intervention, nutrition, internet

## Abstract

**Background:**

Severe obesity in adolescents has deleterious physical and psychological complications necessitating frequent multi-disciplinary clinic visits. Greater treatment engagement has been equated with weight-loss. However, traditional medical weight-loss programs for adolescents have high attrition rates. Social media is widely used by adolescents and may enhance medical weight management engagement and success.

**Objective:**

The first objective was to examine the acceptability and feasibility of using a private social media group as an adjunct to medical weight management in youth ages 14 to 20 years with severe obesity [body mass index (BMI) ≥ 35 kg/m2]. The second objective was to pilot test the use of social media to improve treatment engagement and decrease attrition rates.

**Methods:**

In this single arm, 12 week pre-post study, participants attended individual clinic visits and participated in a moderated private social media group that received nutrition, exercise, and behavior change social media communications or “posts” 3 to 4 times/week. Youth commented and/or liked posts from the moderator and each other. Social media engagement was measured with the number of likes and comments on social media. Clinic attrition was compared, measuring clinic visit attendance 12 weeks prior, during, and after the intervention with mixed linear regression models. Correlations of social media engagement with changes from baseline for BMI, BMI-z score, and psychosocial measures were fit.

**Results:**

All 13 enrolled youth completed the study and reported that the group was enjoyable, helpful, reinforced their weight management program, and would recommend using social media to support other youth. The pilot trial was acceptable and feasible. Youth mean weekly engagement (likes + comments) in social media was greater than once a day (8.6 ±3.6). Compared to 12 weeks prior to the intervention, there was no significant decrease in clinic visit attendance at the end of the intervention (M=.231, *P*=.69) or 12 weeks at the conclusion of the intervention (M=.589, *P*=.28). Increased social media comments correlated with weight change (*r*=–.633, *P*=.04).

**Conclusions:**

This pilot trial demonstrated that the use of social media as an adjunct to medical weight management was feasible and acceptable to adolescents with severe obesity. Based upon these preliminary findings, social media may be an effective way to mitigate attrition from obesity treatment programs, and improve health outcomes in this high-risk population.

## Introduction

Over 3 million youth in the United States (9%) ages 10 to 19 have severe obesity, defined as a body mass index (BMI) ≥ 120% above the 95^th^ percentile or BMI ≥ 35 kg/m^2^ [1,2]. Severe obesity and its associated physical impairments such as type 2 diabetes, obstructive sleep apnea and hypertension require treatment involving frequent follow-up with a multidisciplinary team (ie, dietitian, physical activity specialist, psychologist, physician and nurse) [3-5]. The United States Preventive Services Task Force recommends weekly contact with the weight management team over six months [5]. Challenges such as missing school or work, lack of social support, and frustration with weight-loss success contribute to observed attrition rates up to 82% in pediatric weight management programs [6-10]. Social media may provide an avenue to keep youth motivated and engaged in treatment.

The use of social media as an adjunct to traditional weight management allows for both peer-based and professional support. Social media can provide tailored and immediate feedback as well as practical, informational and peer support, which may be beneficial in childhood obesity treatment [11]. Traditional adolescent obesity treatment programs include parents and families, but generally do not include peers. Adolescent peers influence health-related behavior through modeling, imitation, and social learning [12,13]. Social media is readily accessible with 87% of youth ages 12 to 17 using social media and having internet access [14]. Further, 73% of all teens and 64% of teens with household incomes of ≤ $50,000, have smartphones [14]. Yet there is a paucity of studies examining the use of social media in weight management of children or adolescents with obesity [15]. This pilot study examined the feasibility of using private social media groups in conjunction with traditional weight management to promote obesity treatment engagement and to decrease attrition rates in adolescents with severe obesity. We hypothesized that the peer support social media intervention would improve clinic visit attendance.

## Methods

### Study Design

This single-arm feasibility study employed a pre-post design. We piloted a social media intervention among youth ages 14 to 20 years enrolled in a multi-disciplinary weight management clinic. To assess change in clinic attrition with and without the social media intervention, adolescents were enrolled in social media after they had completed 12 weeks of weight management in a tertiary care center.

### Recruitment

**Figure 1:**
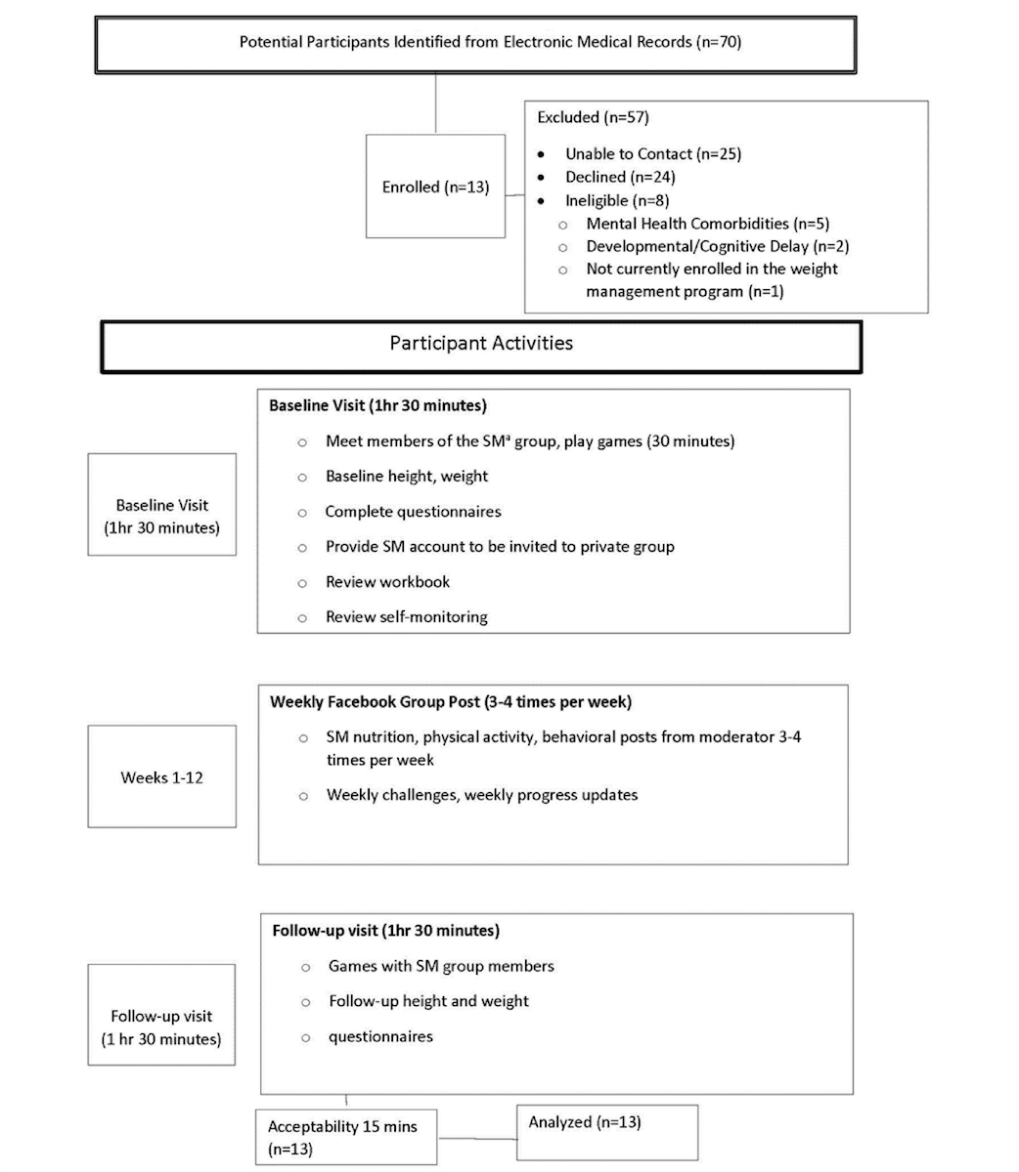
Study Flow diagram. SM=social media.

Participants were recruited from two urban tertiary care centers that treated adolescent obesity. Rosters were generated from the electronic health record of potentially eligible participants. Letters describing the study were sent to the home and participants were contacted and screened by telephone. Additionally, study flyers were placed in clinics. Eligible youth (n=13) were currently enrolled in an outpatient medical weight management program, had a BMI ≥ 35 kg/m^2^, spoke English and had access to either a computer, tablet or a smartphone with a data and text plan. Youth with syndromic or secondary obesity, developmental delay, active substance abuse, untreated depression, psychosis, or an eating disorder other than binge eating disorder were excluded. Secondary to issues of social maturity, youth were divided into two groups based upon age: Group 1 (14 to 16 years) and Group 2 (17 to 20 years). Informed consent was obtained for experimentation with human subjects. The study flow is outlined in Figure 1.

**Enrollment Visit**

Youth were encouraged to attend monthly clinic visits with 1 or 2 medical weight management team members (dietitian, exercise physiologist, physician and psychologist) for 45 minutes and to follow their recommendations. At the enrollment visit, youth met the members of their age-based group and the group moderator, played icebreaker games, were invited to the private social media Facebook group, as well as MyFitnessPal. Acceptance of the invitation to the group was confirmed at the visit. Youth were given an age appropriate treatment manual based upon national guidelines that has previously been tested in adolescents to complement the in-person clinic visits [16,17]. They were advised to consume 1300-1500 kcal/day, to increase daily physical activity to 60 minutes, and to self-monitor these goals daily. Participants completed surveys in person at the enrollment and 12-week follow-up visits.

### Intervention

**Figure 2:**
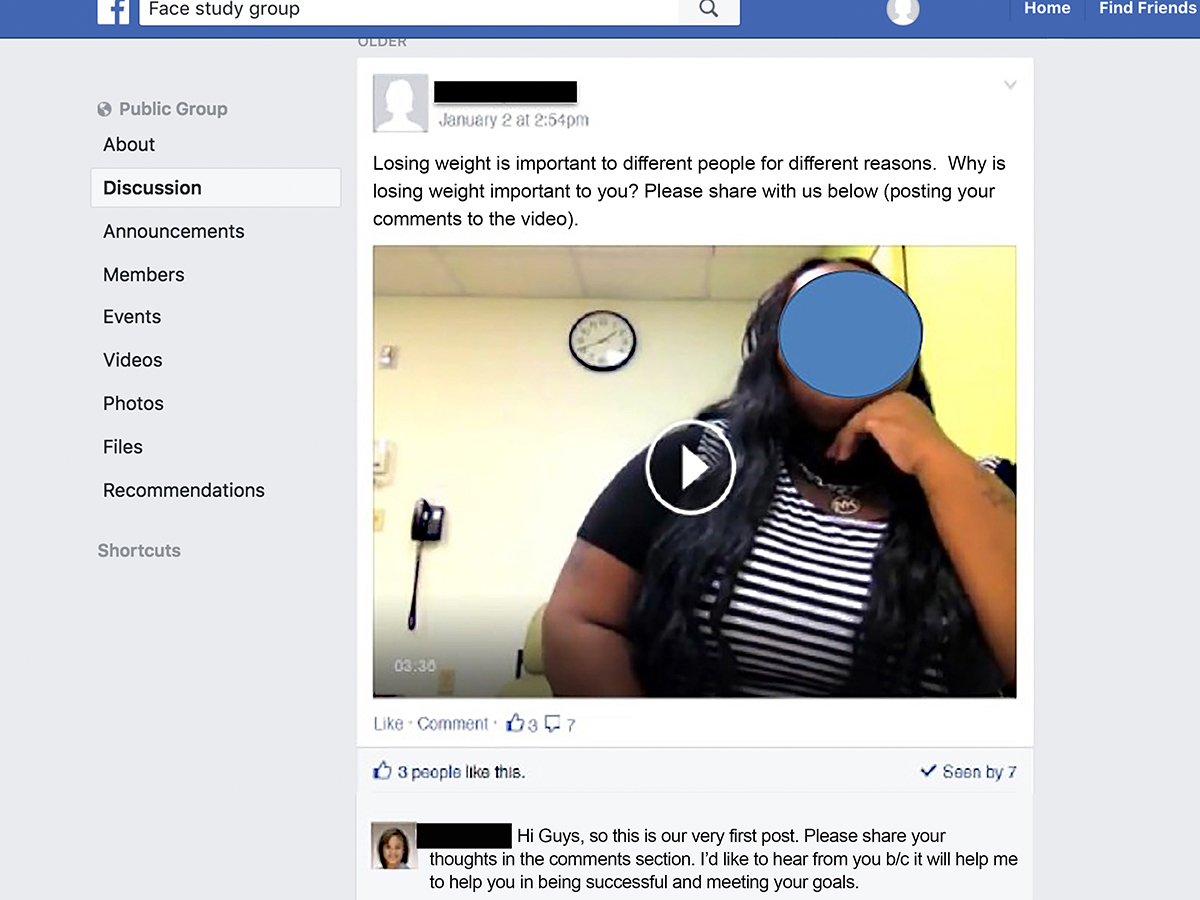
This is the Facebook support page video introduction.

**Figure 3:**
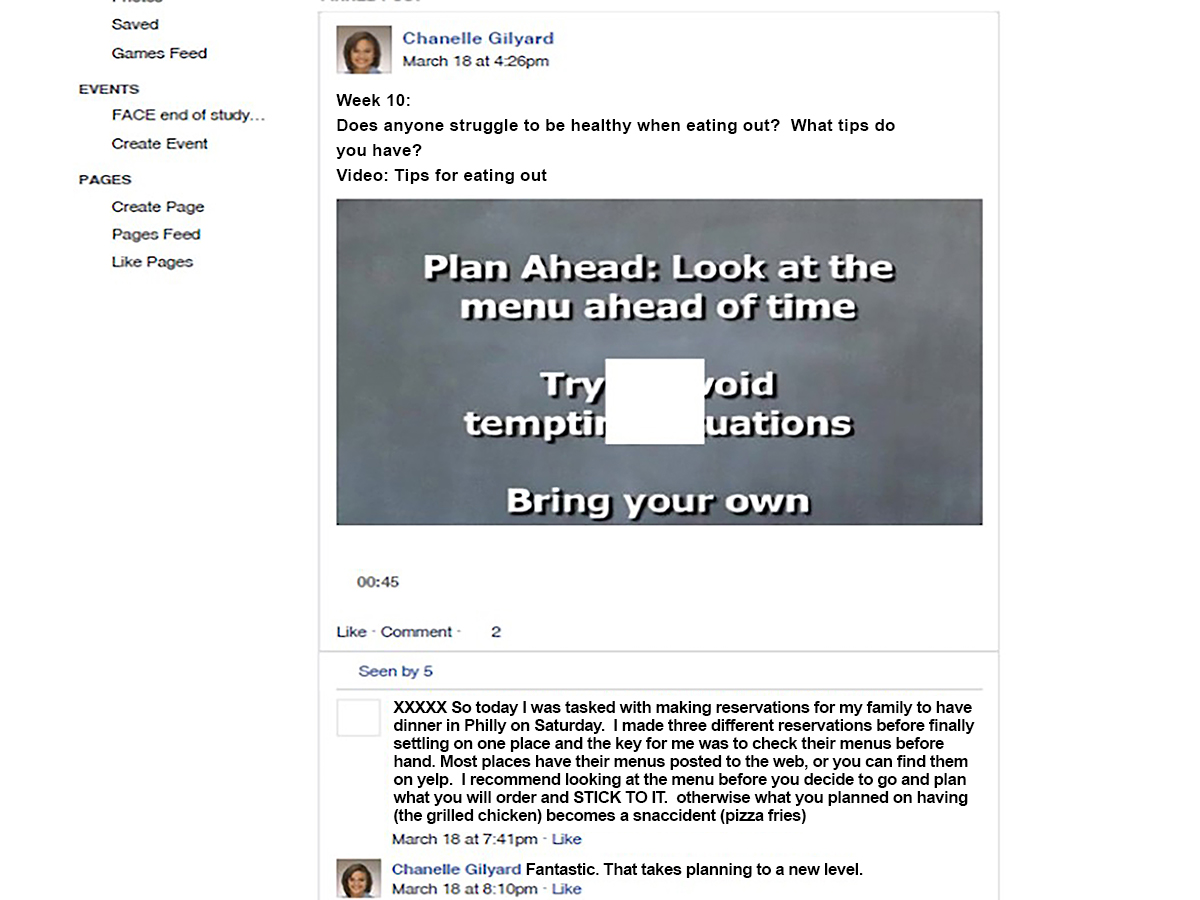
This is a FACE page with prompt and participant response leading to the video.

This 12-week pilot intervention utilized the private social media group moderated by a clinical psychologist. The moderator posted challenges and requests for updates on progress of participants’ goal setting weekly, and posted videos three times each week. Videos featured both youth and “experts” (dieticians, exercise physiologist, physicians, and the psychologist from their weight management team). Video content complemented the treatment manual and included: nutrition, physical activity, behavioral modification topics, cooking, and exercise demonstrations, and youth describing successes and challenges with adopting healthier behaviors (Figures 2 and 3). Youth were encouraged to share their own videos of behaviors and to comment on the moderator and peer posts. The moderator logged in twice a day to check and respond to posts.

As the study involved participants using their personal mobile phones to access the group, each received a US $25 monthly stipend for 3 months to offset the cost of their phone data plan. Youth were informed that in order to be eligible for the stipend, they needed to post, like, or comment in Facebook at least 3 times per week. Beyond those basic guidelines, they were told that they could participate in the group as much or as little as desired. Participants who did not access the group for over 1 week received a private Facebook message with a reminder from the group moderator. Youth were also compensated for two in-person study visits to collect questionnaires and measure height and weight ($25/each). Facebook was chosen as the social media platform because of prior work, and because it was the most popular with youth in our age demographic at the time [14]. A generic name was given to the group “FACE” (Figure 2) that was not health or obesity related. Youth were counselled about the privacy settings. The Children’s Hospital of Philadelphia Internal Review Board was an active participant in insuring participant privacy and protection. Only participants who were invited by the study team could view or post to the group. Participants were able to receive individualized tailored feedback weekly in their social media inbox from the moderator and during their regular clinic appointments. Participants were encouraged to set individual goals, and to post them to the group based upon the weekly challenges and video posts. Participants were encouraged to share feedback on the progress of their goals with the group and to also provide feedback to their peers. They also had the option to inbox the moderator with the progress of their goals, to receive feedback.

### Measures

#### Acceptability and Clinic Attrition

Acceptability of the social media group was assessed using a 28-item questionnaire which consisted of 14 Likert-scale questions and 14 open-response questions adapted from a previously used questionnaire [15]. Families were advised (by their medical team) to come to clinic once a month. Clinic attendances 12 weeks prior, during, and after the intervention were compared. The total clinic visits attended over a 12-week period were calculated to determine attrition. [8].

#### Social Media Engagement

Social media engagement was assessed by compiling the total number of “likes” plus comments [18]. “Likes” and comments were each also examined separately. Participants were incentivized to engage a minimum of 3 times per week in order to watch the 3 video postings. Participants were not considered to be engaged in social media if total engagement was less than or equal to 3 times per week. In exploratory analysis, the effect of social media engagement on BMI, BMI-z-score, depression, quality of life and perceived social support was examined.

#### Anthropometrics

BMI (kg/m^2^) and BMI-z-score were calculated from mean weight (to 0.1 kg) and height (0.1cm), which were measured on a digital electronic scale and wall-mounted stadiometer in triplicate at the enrollment visit and at the end of 12 weeks. Youth changed into scrubs at each measurement visit.

#### Psychosocial measures

*Psychosocial outcomes* included depression, quality of life and perceived social support, which were measured by self-report measures. Depression was measured by using the Beck Depression Inventory II (BDI-II), a 21-item inventory that measures mood with a higher score indicating greater depression [19]. Quality of life was measured with the Impact of Weight on Quality of Life Kids (IWQOL-kids), a 27-item inventory designed to assess the impact of weight status on quality of life, with a higher score indicating a better quality of life [20]. Perceived social support was assessed with the Multidimensional Scale of Perceived Social Support (MSPSS), a 12-item inventory assessing the social support of family, friends, and significant others, with a higher score indicating increased perceived social support [21]. The BDI-II, the IWQOL-kids and the MSPSS items report a test retest reliability of ≥ .78 and a validity score of ≥ .80 in obese adolescents [19,21,22].

#### Statistical Analysis

Descriptive analyses were described using calculated means and standard deviations. For the exploratory analysis of secondary outcomes, Wilcoxon rank sum was used to test changes at 12 weeks. Models were fit correlating mean total engagement (likes + comments), total likes and total comments, and total weekly engagement as a binary variable ≤ or > three times per week and total engagement ≤ or > 36 times total with BMI, BMI-z, depression, quality of life and perceived social support. To test our hypothesis and to assess changes in clinic visit, attendance over time mixed effects analysis were used. Mixed-model analyses allow for the incorporation of proper time trends (eg, nonlinear) and a variance-covariance structure that accounts for the correlation between repeated measures. The mixed-model included the group variable and time. Results are summarized with mean and by time. Analyses were conducted utilizing SAS statistical software (version 9.4; SAS Institute, Cary, NC). For all analyses, an α level of 0.05 was considered statistically significant.

## Results

### Participant Characteristics

Secondary to the limited sample size, results for the combined groups are presented. The majority of youth were female, with private insurance, and had an average BMI of 45 kg/m^2^, which is adult class III extreme obesity, or pediatric severe obesity. There was an almost even racial distribution. The mean age of participants was 16 years of age (Table 1).

### Social Media Usage

Overall, youth remained engaged in social media weekly (likes/comments > 4 times per week), mean 8.6 (SD 3.6). Youth “liked” more than they “commented” (Table 2). The number of times that posts were viewed by participants was unknown.

### Intervention Acceptability

Overall, youth found the social media support group to be enjoyable (100% [13/13]), helpful (100% [13/13]), a source of motivation (100% [13/13]), and would recommend the social media group to other youth with severe obesity (100 [13/13]). Participants felt the group was helpful for peer support (85% [11/13]), advice on nutrition (100% [13/13]), exercise (85% [11/13]), and helpful to reinforce the goals for their weight management program (100% [13/13]). From the open responses, youth identified the group as helping them to be “accountable and feel that they were not alone” and “to receive positive reinforcement.” Youth reported that it was important to have an in-person weight management program along with the social media support group (92% [12/13]). Although adolescents could participate in social media at whatever time was convenient to them, they listed “time constraints of schoolwork and extracurricular activities” as preventing more frequent logins.

### Intervention Feasibility

There were a total of 105 social media contacts from the moderator. Posts from the moderator included videos (63), challenges (6), requests for updates from participants (12), polls (2), pictures and links (9) and other information relevant to the intervention (13). Youth also uploaded videos and pictures (12). The moderator spent approximately 5 hours per week posting material and moderating comments for a total of 60 hours over the course of the 12-week intervention. All participants (n=13) completed the intervention and based upon insurance information included socioeconomically diverse background with 38% (5/13) using Medicaid.

**Table 1:** Participant baseline characteristics (N=13). BMI: body mass index.

Characteristic		Value
Age (years), mean (SD)		16.0 (1.30)
Age (years), range		14-20
Sex (male), n		4
Weight (kg), mean (SD)		127.0 (20.00)
BMI (kg/m^2^), mean (SD)		45.5 (7.30)
BMI-z-score, mean (SD)		2.52 (0.2)
Clinic attendance, mean (SD)		5.85 (4.08)
Race (African American), n		6
Medicaid coverage, n		5
Depression, mean (SD)		7.62 (7.85)
**Quality of life, mean (SD)**		
	Total score	79.64 (12.79)
	Body Esteem subscale score	33.30 (6.94)
	Social Life subscore	27.50 (3.06)
Perceived social support, mean (SD)		74.31 (9.01)

**Table 2:** Descriptive results of social media engagement over the course of 12 weeks (total) and weekly (N=13). Engagement = likes + comments.

Total and weekly engagement	Mean (SD)	Range
Total likes	43.1 (23.5)	9-82
Total comments	12.9 (3-43)	3-18
Total engagement	56 (24.3)	14-85
Weekly engagement	8.6 (3.6)	14-85

**Table 3:** Spearman Correlation of social media engagement with change in secondary outcomes in all subjects (N=13). BMI: body mass index.

Secondary outcomes	Total likes		Total comments		Total likes + comments	
	*r*	*P* value	*r*	*P* value	*r*	*P* value
Weight (kg)	.463	.11	–.724	.005	.138	.65
BMI (kg/m^2^)	.275	.36	–.550	.05	.008	.98
BMI z-score	.252	.43	–.532	.08	–.053	.87
Depression^a^	.277	.36	–.084	.78	.064	.84
Quality of life^b^	–.119	.71	383	.22	–.077	.81
Perceived social support^c^	.204	.50	.198	.51	.044	.97

^a^Depression measured by the Beck Depression Inventory II (higher=worse depression).

^b^Quality of life measured by the Impact of Weight on Quality of Life—Kids (higher=better quality of life).

^c^Perceived social support measured by Multidimensional Scale of Perceived Social (higher=better social support)

### Clinic Attrition

In mixed effects analysis, we included all subjects controlling for group to evaluate change in clinic attendance with time. The number of visits prior to the start of the intervention (baseline) were compared at 12 weeks (the end of the intervention) mean (M) =.231, *P*=.69, and 6 months (3 months at the conclusion of the intervention) (M=.589, *P*=.28). There was no significant change in clinic visit attendance across time points. This indicates there was not an increase in clinic attrition. Given high attrition rates from adolescent obesity treatment programs, this preliminary finding of continued engagement in treatment is encouraging [9].

### Social Media Engagement

All analyses were exploratory and conducted in the combined sample controlling for group effect, secondary to the sample size. Mean changes were observed at 12 weeks for all participants for weight in kilograms (odds ratio [OR] –1.01 [95% CI –6.1, 4.08]), BMI (OR –1.25 [CI –2.99, 0.49]), BMI-z-score (OR –0.03 [CI –0.1, 0.03]), depression ( OR –1.69 [CI –4.96, 1.57]), quality of life (OR 3.84 [CI –2.63, 10.31]) and perceived social support (OR 3.46 [CI –1.06, 7.99]). Greater numbers of comments correlated with weight-loss at 12 weeks (*r*=–.633, *P*=.04) (Table 3).

## Discussion

### Principal Findings

All enrolled adolescents (n=13) completed this social media intervention and found the use as an adjunct to medical weight management feasible and acceptable. Adolescents enjoyed the social media group, found it that it kept them motivated, encouraged, accountable, and informed. More than half of the group wanted to have more in-person groups as part of an intervention. Participants remained engaged in the social media group and within the in-person clinic. Clinic attrition decreased during the intervention. Youth remained engaged in the social media group overall with more likes than comments. Increased engagement with comments was correlated with weight-loss and increased perceived social support. We developed a privacy and safety plan that met the requirements of the institutional review board, which included the use of a private/secret group and monitoring/screening of posts.

### Comparison with Prior Work

Compared with prior studies, our pilot demonstrated lower clinic visit attrition rates (27%) vs 42-82% [6-8]. The addition of a social media support group addressed key factors identified by adolescents for attrition from obesity treatment: a lack of peer support, logistical challenges, poor expectations, and a lack of tailoring of treatment for adolescents [7,23]. Participants confirmed the role of social media on clinic attendance in our acceptability survey in which they indicated that the social media group influenced their clinic participation, motivation and compliance with behavior changes.

Despite the small size of our pilot group, the overall engagement in social media was high at 71% compared with studies using social media alone in older adolescents (41%) [11,18]. In order to achieve higher rates of engagement, we incorporated successful strategies from prior studies such as having an in-person group session for participants to meet one another at the beginning of the intervention, the use of a facilitator who provided constant reinforcement of participation according to Social Learning Theory, and utilized a “secret” group for privacy [15,24]. While polls were the most popular type of post in a study examining types of posts in adults, videos were the most popular post in this study [18]. Videos also included other adolescents, which may have encouraged participation. [25] Also similar to prior work we provided a US $25 monthly incentive to assist with the cost of mobile data for active participants [15]. Participants were incentivized for 3 posts and did not receive any additional incentives. Participant engagement was more than twice the incentivized amount (weekly mean=8.6 compared with requested 3 times per week). Additionally, youth were not incentivized to attend clinic visits.

To our knowledge, this is the first study to examine the influence of a social media support group on medical weight management engagement/attrition. Attrition from in-person clinic visits did not increase in our study compared to rates of 50% or more normally seen in this population. Our use of age-based adolescent peer groups may address attrition by providing adolescent tailored interventions and increasing perceived peer support [26]. On the acceptability survey, participants indicated that the social media group influenced their clinic participation and reinforced what they learned in their medical weight management (100% [13/13]).

### Inclusion of Parents/Family Members

Finding ways to incorporate parents/caregivers into the social media component will be important in next steps. Parents purchase the food for the home, provide transportation and financial support for physical activity, serve as role models of behavior and provide encouragement and support for behavioral modification [27-29]. In this study, parents attended clinic sessions with adolescents where goal setting was made at both the family and individual levels. During baseline and 12-week follow-up visits, parents of participants remained in a separate room to get to know one another. Parents expressed interest in a parent social media group. As parents are key agents of change, providing peer support on the parent level may also increase adolescent clinic attendance and should be explored in next steps.

### Limitations and Strengths

Our study has both strengths and limitations. This single arm pilot study had a small sample size to determine feasibility. All enrolled participants completed this pre-post study. Although our sample size was small, it was racially and socioeconomically diverse. Heights and weights were objectively measured. Both quantitative and qualitative assessments of acceptability were used. Despite the small sample size, some significant associations were detected.

However, as this is a cross-sectional study, the associations found do not necessarily reflect causality. Furthermore, it is possible that participants in this sample had a higher level of intrinsic motivation which kept them engaged. The data from this pilot trial will inform the conduct of a larger randomized controlled clinical trial. Adolescents were incentivized to like or comment which could have increased social media engagement. Incentives did not increase for liking vs commenting, or for engagement above the required 3 times per week. Engagement was above the minimum requirement for incentivization. While the majority of peer support came from social media, we acknowledge that the two in-person visits (for measurements and to meet other group members) could have also influenced perceived social support. That being said, adolescents were not incentivized to attend clinic visits. Our findings cannot be extrapolated to other forms of social media. Facebook was the most popular form of social media in youth at the time of our study [13]. Although, not as popular with youth today, Facebook is still one of the most popular social media platforms used by adolescents [14]. That being said, other platforms should also be tested.

Other studies have found that social media engagement decreases over time [18,30]. It will be important to test the effects of a social media group longer term on attrition. Similar to prior studies, we measured engagement as a combination of likes and comments [18]. We acknowledge that commenting most likely reflects a higher level of engagement than liking as it requires reflection. This is also reflective of increased commenting as opposed to liking being associated with weight-loss. Finding ways to assist youth with interacting through comments is important for future interventions.

## Conclusions

In summary, youth with severe obesity found social media support acceptable and useful as an adjunct to clinical care, and perceived it as helpful to remain motivated to attend medical weight management. Pilot results suggest social media groups may assist with decreasing clinic attrition rates and potentially contribute to successful weight management in this high-risk population.

## Abbreviations

BDI-II: Beck Depression Inventory II
BMI: body mass index
IWQOL-kids: Impact of Weight on Quality of Life Kids
MSPSS: Multidimensional Scale of Perceived Social Support
OR: odds ratio
CHOP: The Children’s Hospital of Philadelphia
